# Analysis of Main Error Sources for the Error Motion Measurement of a Precision Shafting Using a T-Type Capacitive Sensor

**DOI:** 10.3390/mi13020221

**Published:** 2022-01-29

**Authors:** Kui Xiang, Wen Wang, Zichen Chen

**Affiliations:** 1 Key Laboratory of Advanced Manufacturing Technology of Zhejiang Province, School of Mechanical Engineering, Zhejiang University, Hangzhou 310027, China; 11125034@zju.edu.cn (K.X.); chenzc@zju.edu.cn (Z.C.); 2 School of Mechanical Engineering, Hangzhou Dianzi University, Hangzhou 310018, China

**Keywords:** precision shafting, capacitive sensor, error sources analysis

## Abstract

As a key indicator reflecting the working accuracy of rotary functional units, the error motions of the precision shafting are very necessary to be measured. In this paper, the main error sources for the error motion measurement of a precision shafting using a T-type capacitive sensor were investigated. The theoretical modeling error due to the approximate simplification for the output capacitance expressions was firstly analyzed. By means of the 3D-FEA method, the influence of fringe effects was subsequently investigated. Finally, the analysis of electrode installation errors was emphasized on the tilt error of the cylindrical electrode and coaxiality error of the fan-shaped electrode by establishing mathematical models and numerical simulation. Based on the theoretical analysis and simulation results, the methods of decreasing the approximate error and the nonlinear error caused by fringe effects were subsequently proposed; for the installation errors, the tilt error of cylindrical electrode only makes the solution of phase angle have a certain deviation and has almost no effect on solving the radial displacement, especially for the measurement range less than 0.1 mm; the measurement of the rotor tilt displacement was basically not affected by the coaxiality error of the fan-shaped electrode.

## 1. Introduction

The rotary functional units with precision shafting as the core are widely used in high-end equipment such as high-precision CNC machine tools, EUV lithography equipment, axial compressors and small agile satellites [1,2,3,4,5,6,7]. As a key indicator reflecting the working accuracy of rotary functional units, the error motions of the precision shafting are very necessary to be measured. This measurement provides valuable insight into the error motions and performance [8], which is favorable to optimize these rotary units.

Capacitive sensors with good stability, high speed and adaptability of extreme environments are widely adopted to measure the displacement of motion targets [9,10,11,12]. Chapman et al. carried out research on the measurement of rotary axis error motions using a cylindrical capacitive sensor (CCS) with multiple electrodes of large sense area [13,14,15]. In order to measure the multi-degree-of-freedom (multi-DOF) error motions, several separate electrodes need to be manufactured and orderly mounted along with the guard ring, which is difficult.

**Figure 1:**
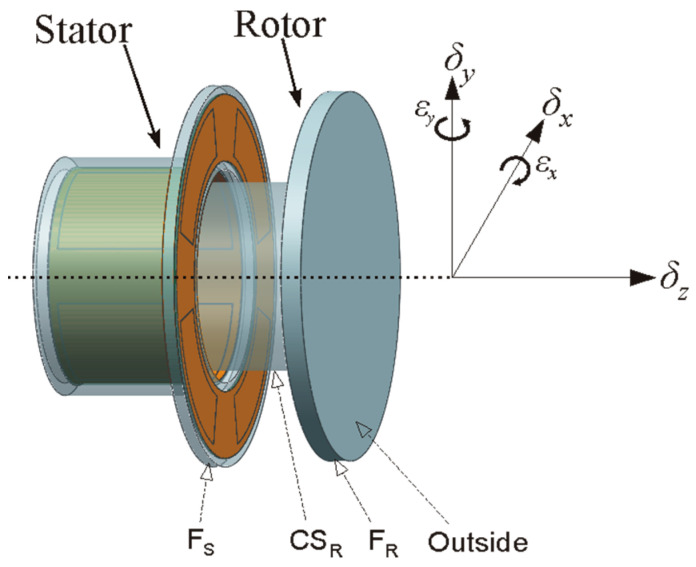
Schematic of a T-type CS. The error motions *δ_x_* and *δ_y_* are detected using the REG, which is in the annular groove of the stator cylindrical bore; *δ_z_* and *ε_x_* and *ε_y_* are detected using the EPEG, which is in the annular groove of the stator end part.

**Figure 2:**
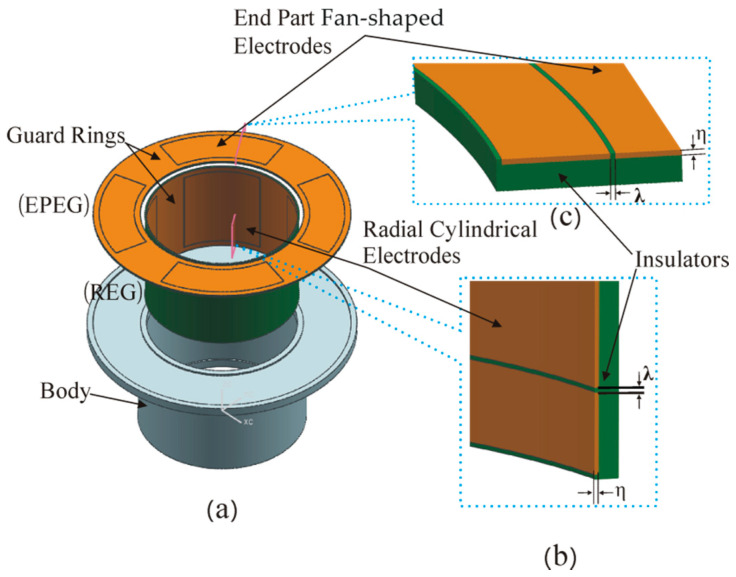
Schematic of the stator: (**a**) The exploded view of the structure; (**b**,**c**) partial cross-sectional view of the stator. *η* is the thickness of the electrode, and *λ* is the gap between the electrode and guard ring.

Compared to the CCS used in the above research, a novel T-type capacitive sensor (T-type CS) with an integrated structure was developed to achieve the simultaneous online measurement of the 5-DOF error motions of a spindle, as shown in Figure 1 and Figure 2 [16]. Both the radial electrode group (REG) and the end part electrode group (EPEG) were fabricated based on a flexible printed circuit board, which could produce a quite thinner electrode (0.035 mm) and a tiny gap (0.15 mm) between the electrode and guard ring.

**Figure 3:**
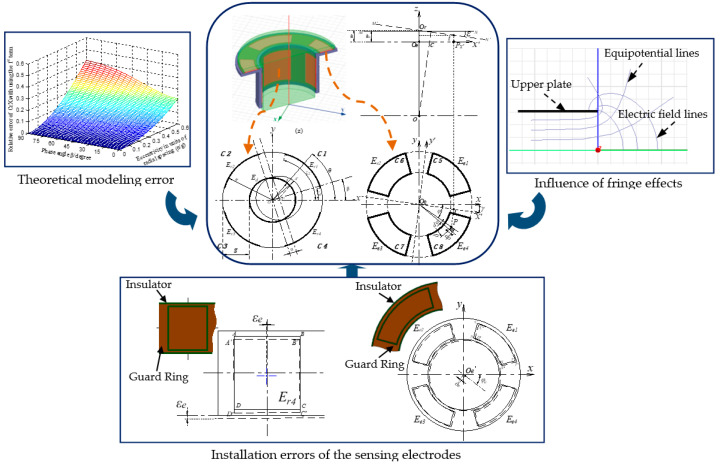
The general sketch of the main research content.

This paper focuses on the analysis of main error sources for the error motion measurement of a precision shafting using a T-type CS, as shown in Figure 3. Firstly, the theoretical modeling error due to the approximate simplification for the output capacitance expression of sensing electrodes was analyzed. Then, the influence of fringe effects was investigated using the 3D-FEA method. Finally, mathematical modeling and numerical simulation were conducted to analyze electrode installation errors, emphasizing the tilt error of the cylindrical electrode and coaxiality error of the fan-shaped electrodes.

## 2. Basic Principle

### 2.1. Radial Error Motion Measurement

**Figure 4:**
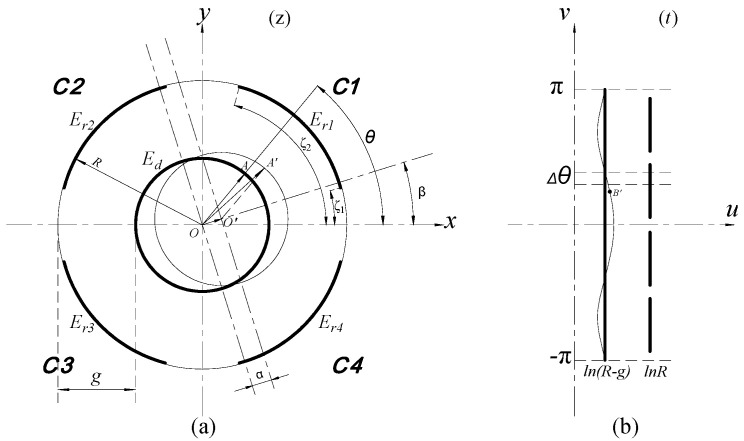
The calculation model of the radial error motions: (**a**) Boundary before transformation; (**b**) boundary after transformation.

This measurement is performed by the REG, which is constituted by four cylindrical electrodes *E_r_*_1_~*E_r_*_4_. The four cylindrical electrodes and the radial measuring surface (CS_R_) of the rotor make up four cylindrical capacitors, respectively; the corresponding capacitance are denoted by C1~C4, as shown in Figure 4 [16].

Due to the arc-shaped boundary of these electrodes, the conformal mapping method was adopted to solve the Poisson Equation in electrostatic fields [17]. When the rotor has radial displacement with eccentricity *α* at phase angle *β*, caused by the generated radial run-out of a spindle, the resulting variation Δ*u* of the equivalent spacing at any point in the complex plane *t* can be approximately derived as [16]:

(1) $$\Delta u=\frac{\alpha \cos(\theta -\beta )}{R}$$

where *R* is the inside radius of the cylindrical electrode, *θ* is the argument of the complex number corresponding to any point on the arc-shaped boundary in the plane *z*.

Thereby, the differential expression of the output capacitance of a cylindrical electrode with axial width of *w* can be illustrated as [16]:

(2) $$\Delta C_{r}=\varepsilon_{0}\varepsilon_{r}\frac{R\Delta \theta w}{g-\alpha \cos\left(\theta -\beta \right)}$$

where *ε*_0_ is the electric permittivity of vacuum, *ε_r_* is the relative dielectric constant; *g* is the initial radial spacing and Δ*θ* is an equivalent length of a micro-unit that is taken on the equivalent boundary in the plane *t*.

Then the output capacitance of the cylindrical electrode in each quadrant is approximately expressed as follows [16]:

(3) $$C1=\varepsilon_{0}\varepsilon_{r}Rw\int_{\zeta_{1}}^{\zeta_{2}}\frac{1}{g-\alpha \cos\left(\theta -\beta \right)}d\theta$$

(4) $$C2=\varepsilon_{0}\varepsilon_{r}Rw\int_{\pi -\zeta_{2}}^{\pi -\zeta_{1}}\frac{1}{g-\alpha \cos\left(\theta -\beta \right)}d\theta$$

(5) $$C3=\varepsilon_{0}\varepsilon_{r}Rw\int_{\pi +\zeta_{1}}^{\pi +\zeta_{2}}\frac{1}{g-\alpha \cos\left(\theta -\beta \right)}d\theta$$

(6) $$C4=\varepsilon_{0}\varepsilon_{r}Rw\int_{2\pi -\zeta_{2}}^{2\pi -\zeta_{1}}\frac{1}{g-\alpha \cos\left(\theta -\beta \right)}d\theta$$

where (*ζ*_2_ − *ζ*_1_) denotes the angular size of the cylindrical electrode.

Based on Equations (3)–(6), the expressions of the differential output capacitance of the REG can be expressed by:

(7) CrX=C1+C4−C2−C3

(8) CrY=C1+C2−C3−C4

where CrX and CrY, respectively, correspond to the components *δ_x_* and *δ_y_* of the displacement *α* in the *X* and *Y* directions.

Then, the radial error motions of the spindle can be estimated by the following equations:

(9) $$\delta_{x}=f_{x}\left(C1+C4-C2-C3\right)$$

(10) $$\delta_{y}=f_{y}\left(C1+C2-C3-C4\right)$$

where *f_x_* and *f_y_* are denoted the transition functions between the measured capacitance of the cylindrical electrodes and the radial run-out along the *x*, *y* directions, respectively.

### 2.2. Axial Error Motion Measurement

**Figure 5:**
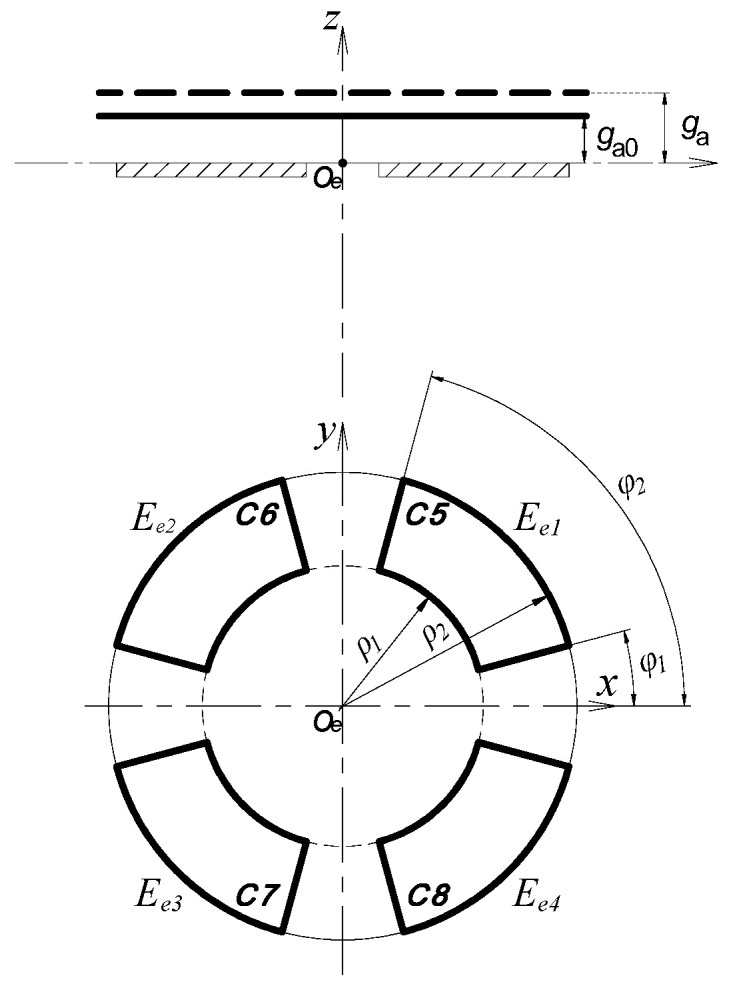
The calculation model of the axial error motion.

As shown in Figure 2, the fan-shaped electrodes (*E_e_*_1_~*E_e_*_4_) of the EPEG are also distributed in the same configuration as radial electrodes. They, along with the inside annular end face of the rotor flange (F_R_), make up four capacitors, respectively; the corresponding capacitance of them are denoted by C5~C8, as shown in Figure 5 [16].

When the rotor has an axial error motion along the *z*-axis, the variation quantity of the sum of the output capacitance of the fan-shaped electrode is given by [16]:

(11) $$\begin{array}{l} \Delta \mathrm{CeZ}={\mathrm{CeZ}}^{\prime }-\mathrm{CeZ}\\ \;\;\;\;\;\;\;\;=4\varepsilon_{0}\varepsilon_{r}S_{e}(\frac{1}{g_{a0}+\Delta z}-\frac{1}{g_{a0}}) \end{array}$$

where *S_e_* is the area of the fan-shaped electrode, *g_a_*_0_ is the initial value of the axial spacing, the variation of the axial spacing Δ*z* is defined by Δ*z* = *g_a_* − *g_a_*_0_, CeZ is the sum of the output capacitance of the fan-shaped electrodes under the axial spacing *g_a_*_0_ and CeZ′ is that of the fan-shaped electrodes under the axial spacing *g_a_*.

According to Equation (11), the expressions for estimating the axial error motion of the spindle can be derived as:

(12) $$\delta_{z}=f_{z}\left(C5+C6+C7+C8\right)$$

### 2.3. Tilt Error Motion Measurement

**Figure 6:**
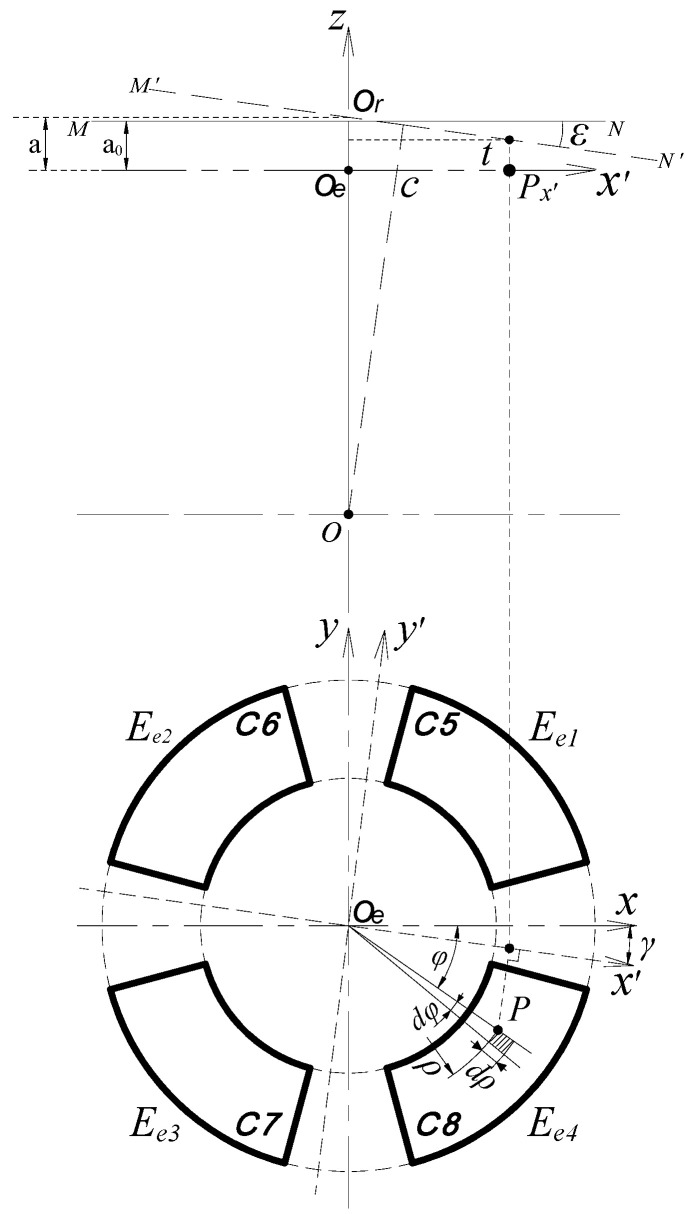
The calculation model of the tilt error motions.

The calculation model for the measurement of tilt error motions by EPEG is shown in Figure 6 [16]. The line segment *MN* represents the axial measuring surface of the rotor (i.e., the inside annular end face of the rotor flange), and its perpendicular bisector is the medial axis of the rotor.

In the boundary region of the fan-shaped electrode, a micro-plane element ΔB_P_ at any point *P* is taken as the solving unit (the area of it is denoted as Δ*S_P_*). The micro-plane element ΔB_P_ and its opposite counterpart ΔA_Q_ compose a plane parallel capacitor (ΔA_Q_ is in the axial measuring surface of the rotor), and the spacing between them is derived as [16]:

(13) t=a−k⋅ρcos(φ−γ)

where *a* is the distance from the intersection point *O_r_* to the EPEG; *k* = tan(*ε*), *ε* and *γ* are the amplitude and yaw angle of the tilt displacement of the rotor, respectively.

Thereby, the capacitance of the plane parallel capacitor is [16]:

(14) $$\Delta C_{e}=\frac{\varepsilon_{0}\varepsilon_{r}\Delta S_{P}}{t}=\varepsilon_{0}\varepsilon_{r}\frac{\rho d\varphi \cdot d\rho }{a-k\cdot \rho \cos(\varphi -\gamma )}$$

Then, the output capacitance of the fan-shaped electrode *E_e_*_4_ can be approximately obtained by integrating Equation (14) over the area of the fan-shaped electrode [16]:

(15) $$C8=\varepsilon_{0}\varepsilon_{r}\int_{{2\pi -\varphi }_{2}}^{{2\pi -\varphi }_{1}}\int_{\rho_{1}}^{\rho_{2}}\frac{\rho d\rho }{a-k\cdot \rho \cos(\varphi +\gamma )}d\varphi$$

where (*φ*_2_ − *φ*_1_) are the angular size of each fan-shaped electrode, and *ρ*_1_ and *ρ*_2_ are the inside and outside radius of the electrode, respectively.

By considering the minor tilt displacement of a precision spindle, the power series expansion by ignoring higher-order terms was applied to express the Equation (15) (*k* << *a*):

(16) $$C8=\frac{\varepsilon_{0}\varepsilon_{r}}{a}\int_{{2\pi -\varphi }_{2}}^{{2\pi -\varphi }_{1}}\left[b+\frac{kc}{a}\cos(\varphi +\gamma )\right]d\varphi$$

where the factors *b* and *c* are given by $b=\frac{\rho_{2}^{2}-\rho_{1}^{2}}{2},\;c=\frac{\rho_{2}^{3}-\rho_{1}^{3}}{3}$.

By analogy with the electrode *E_e_*_4_, the output capacitance of the other electrodes can be approximately expressed as follows:

(17) $$C7=\frac{\varepsilon_{0}\varepsilon_{r}}{a}\int_{{\pi +\varphi }_{1}}^{{\pi +\varphi }_{2}}\left[b+\frac{kc}{a}\cos(\varphi +\gamma )\right]d\varphi$$

(18) $$C6=\frac{\varepsilon_{0}\varepsilon_{r}}{a}\int_{{\pi -\varphi }_{2}}^{{\pi -\varphi }_{1}}\left[b+\frac{kc}{a}\cos(\varphi +\gamma )\right]d\varphi$$

(19) $$C5=\frac{\varepsilon_{0}\varepsilon_{r}}{a}\int_{\varphi_{1}}^{\varphi_{2}}\left[b+\frac{kc}{a}\cos(\varphi +\gamma )\right]d\varphi$$

By making an arrangement, Equations (16)–(19) are given as:

(20) $$C8=\frac{\varepsilon_{0}\varepsilon_{r}}{a}\int_{\varphi_{1}}^{\varphi_{2}}\left[b+\frac{kc}{a}\cos(\varphi -\gamma )\right]d\varphi$$

(21) $$C7=\frac{\varepsilon_{0}\varepsilon_{r}}{a}\int_{\varphi_{1}}^{\varphi_{2}}\left[b-\frac{kc}{a}\cos(\varphi +\gamma )\right]d\varphi$$

(22) $$C6=\frac{\varepsilon_{0}\varepsilon_{r}}{a}\int_{\varphi_{1}}^{\varphi_{2}}\left[b-\frac{kc}{a}\cos(\varphi -\gamma )\right]d\varphi$$

(23) $$C5=\frac{\varepsilon_{0}\varepsilon_{r}}{a}\int_{\varphi_{1}}^{\varphi_{2}}\left[b+\frac{kc}{a}\cos(\varphi +\gamma )\right]d\varphi$$

By combining Equations (20)–(23), the distance *a* can be derived as:

(24) $$a=\frac{4\varepsilon_{0}\varepsilon_{r}b\cdot (\varphi_{2}-\varphi_{1})}{C8+C7+C6+C5}$$

Based on Equations (16)–(19), the expressions of the differential output capacitance of the EPEG can be expressed as follows:

(25) CeX=C5+C6−C7−C8

(26) CeY=C5+C8−C6−C7

where CeX and CeY, respectively, correspond to the components *ε_x_* (around the *x*-axis), *ε_y_* (around the *y*-axis) of the tilt displacement *ε*.

Then, the tilt error motions of the spindle can be estimated by the following equations:

(27) $$\varepsilon_{x}=f_{ex}\left(C5+C6-C7-C8\right)$$

(28) $$\varepsilon_{y}=f_{ey}\left(C5+C8-C6-C7\right)$$

where *f_ex_* and *f_ey_* are denoted as the transition functions between the measured capacitance of the fan-shaped electrodes and the tilt displacements about the *x*- and *y*-axis, respectively.

## 3. Main Error Sources Analysis of the T-Type CS

### 3.1. Theoretical Modeling Error Analysis

In order to theoretically analyze the radial error motion measurement with the T-type CS, we consider a small radial displacement of the precision spindle, i.e., |*α*/(*R* − *g*)| < 1. The power series expansion with first-order approximation (Equation (1)) was applied to express the equivalent spacing changing Δ*u*. The exact derived expression of it is:

(29) $$\Delta u_{o}=\frac{1}{2}\ln\left\{\frac{{\left(R-g\right)}^{2}+2\alpha (R-g)\cos(\theta -\beta )+\alpha^{2}}{{\left(R-g\right)}^{2}}\right\}$$

Then, the approximate error of Equation (1) can be assessed by:

(30) $$\Delta u_{app.error}=\left|\frac{\Delta u_{o}-\Delta u}{\Delta u_{o}}\right|$$

**Figure 7:**
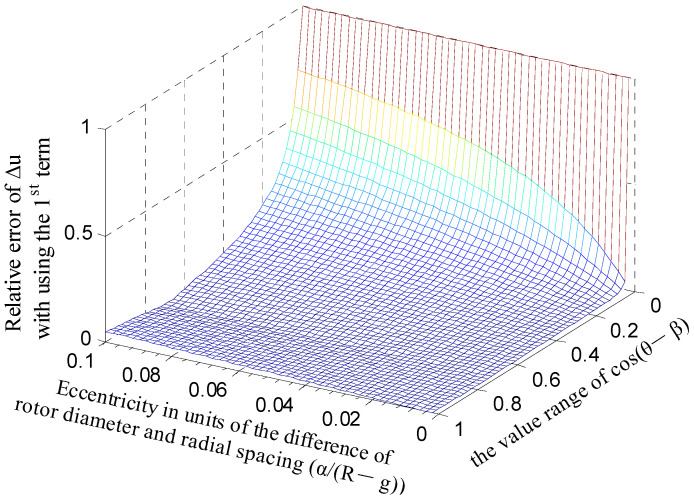
Error distribution of the approximate calculation of the changing Δ*u* in the complex plane *t*.

Figure 7 shows the variation situation of error Δ*u_app.error_* with the changing of parameter terms *α*/(*R* − *g*) and cos(*θ* − *β*). As mentioned above, the radial displacement *α* of a precision spindle is generally very small. In order to achieve relatively high sensitivity, the cylindrical electrode radius *R* is often designed to be tens of millimeters or even larger. Thus, the variation range of the term *α*/(*R* − *g*) is set to be [0, 0.1]. It can be observed that, when the ratio *α*/(*R* − *g*) is less than 0.01, the error Δ*u_app.error_* basically keeps lower than 2% with the variation in the term cos(*θ* − *β*) in the entire phase region (the value range of cos(*θ* − *β*) is [0, 1] correspondingly), except for a few phase angles. Therefore, a highly accurate value of the changing Δ*u* calculated by Equation (1) can be obtained if the ratio *α*/(*R* − *g*) is less than 0.01, which can be achieved by adjusting the structural parameters of the sensor.

Since a highly accurate value of Δ*u* can be obtained by setting the ratio *α*/(*R − g*) ≤ 0.01, the expressions (3)–(6) established based on Equation (1) could be more accurate to reflect the capacitance changing caused by the radial displacement, and is also for the expressions of the differential output capacitance of the REG derived as:

(31) $$\mathrm{CrX}=\frac{2\varepsilon_{0}\varepsilon_{r}Rw}{g\sqrt{1-{\left(\frac{\alpha }{g}\right)}^{2}}}\left(\arctan\frac{2\frac{\alpha }{g}\sqrt{1-{\left(\frac{\alpha }{g}\right)}^{2}}\sin\zeta_{2}\cos\beta }{1-{\left(\frac{\alpha }{g}\right)}^{2}\left(\cos^{2}\beta +\sin^{2}\zeta_{2}\right)}-\arctan\frac{2\frac{\alpha }{g}\sqrt{1-{\left(\frac{\alpha }{g}\right)}^{2}}\sin\zeta_{1}\cos\beta }{1-{\left(\frac{\alpha }{g}\right)}^{2}\left(\cos^{2}\beta +\sin^{2}\zeta_{1}\right)}\right)$$

(32) $$\mathrm{CrY}=\frac{2\varepsilon_{0}\varepsilon_{r}Rw}{g\sqrt{1-{\left(\frac{\alpha }{g}\right)}^{2}}}\left(\arctan\frac{2\frac{\alpha }{g}\sqrt{1-{\left(\frac{\alpha }{g}\right)}^{2}}\cos\zeta_{1}\sin\beta }{1+{\left(\frac{\alpha }{g}\right)}^{2}\left(\cos^{2}\beta +\sin^{2}\zeta_{1}-2\right)}-\arctan\frac{2\frac{\alpha }{g}\sqrt{1-{\left(\frac{\alpha }{g}\right)}^{2}}\cos\zeta_{2}\sin\beta }{1+{\left(\frac{\alpha }{g}\right)}^{2}\left(\cos^{2}\beta +\sin^{2}\zeta_{2}-2\right)}\right)$$

In a manner similar to the simplification process on the equivalent spacing changing Δ*u*, the first-order approximation of the Equations (31) and (32) could intuitively reflect the variation’s relation of the radial displacement and the differential capacitance (CrX and CrY). Thus, the expressions of the displacement *α* and phase angle *β* can be deduced in the following form:

(33) $${\mathrm{CrX}}_{\mathrm{tl}}=\frac{4\varepsilon_{0}\varepsilon_{r}Rw(\sin\zeta_{2}-\sin\zeta_{1})}{g^{2}}\cdot \alpha \cos\beta$$

(34) $${\mathrm{CrY}}_{\mathrm{tl}}=\frac{4\varepsilon_{0}\varepsilon_{r}Rw(\cos\zeta_{1}-\cos\zeta_{2})}{g^{2}}\cdot \alpha \sin\beta$$

Therefore, the expression used to assess the approximate error (taking the CrX as an example) is given as:

(35) $${\mathrm{CrX}}_{app.error}=\left|\frac{\mathrm{CrX}-{\mathrm{CrX}}_{\mathrm{tl}}}{\mathrm{CrX}}\right|$$

**Figure 8:**
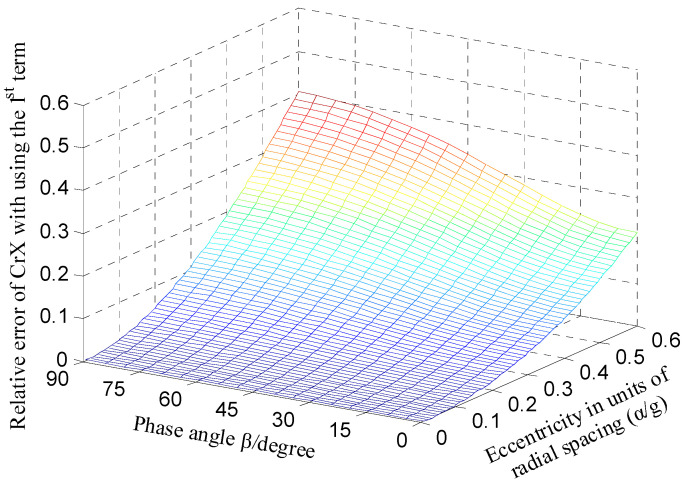
Calculation error distribution of CrX (expansion with first-order approximation).

**Figure 9:**
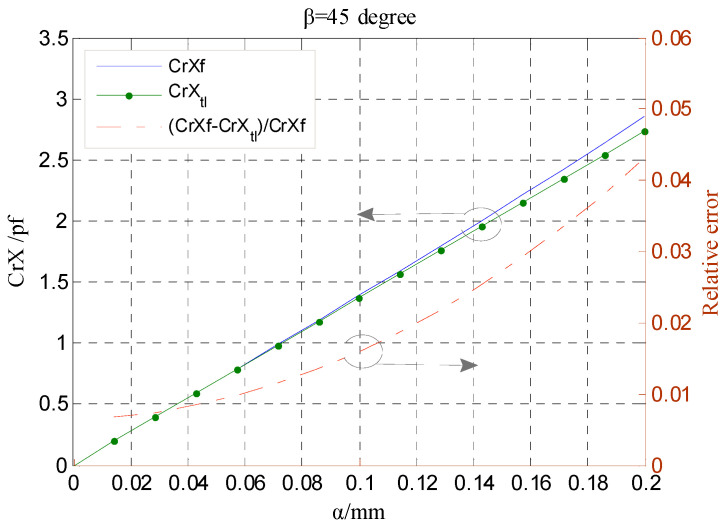
Simulation values and theoretical one calculated by Equation (33) comparison of radial differential output capacitance.

As shown in Figure 8, at each phase angle *β*, the approximate error CrX*_app.error_* all increases with the increase in the ratio *α*/*g*. When the ratio *α*/*g* becomes larger than 0.2, the variation tendency is more significant. Figure 9 shows the error of the calculated capacitance CrX_tl_ relative to the simulation value CrXf as the rotor has a displacement along the direction of y = x. The dependence of the relative error on the parameter *α* is basically consistent with the variation tendency shown in Figure 8. Note that the value CrXf was obtained based on the simulation model of the T-type CS previously established with the design parameters in [16]; these parameters were also adopted to calculate the theoretical capacitance.

From the analysis in Figure 8, it can be known that diminishing the ratio *α*/*g* could make the error CrX*_app.error_* reduce further, but the measurement range and sensitivity of the sensor will be affected to some extent. By considering the inherent nonlinearity of the capacitor output with spacing change, the power series expansion with a third-order approximation was derived to improve the calculation accuracy [18]:

(36) $${\mathrm{CrX}}_{\mathrm{tl}3}=\frac{4\varepsilon_{0}\varepsilon_{r}Rw(\sin\zeta_{2}-\sin\zeta_{1})}{g}\cdot \left(\frac{\alpha }{g}+0.95{\left(\frac{\alpha }{g}\right)}^{3}\right)\cdot \cos\beta$$

(37) $${\mathrm{CrY}}_{\mathrm{tl}3}=\frac{4\varepsilon_{0}\varepsilon_{r}Rw(\cos\zeta_{1}-\cos\zeta_{2})}{g}\cdot \left(\frac{\alpha }{g}+0.95{\left(\frac{\alpha }{g}\right)}^{3}\right)\cdot \sin\beta$$

**Figure 10:**
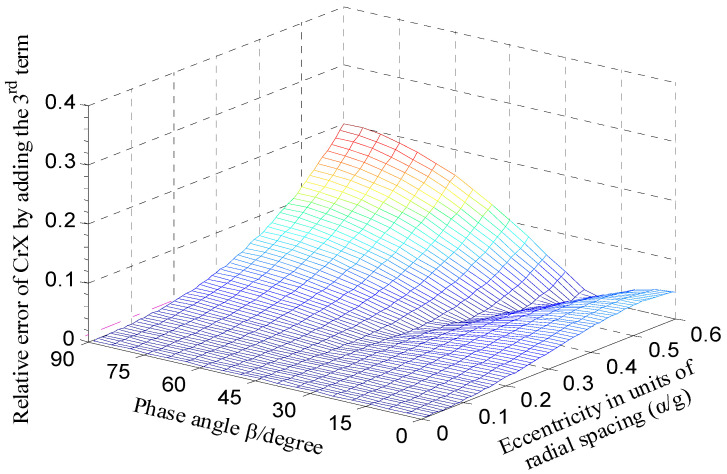
Calculation error distribution of CrX (expansion with third-order approximation).

**Figure 11:**
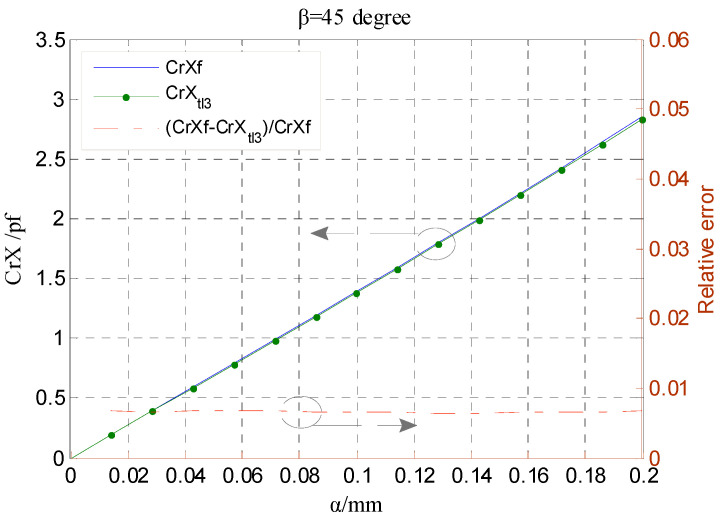
Simulation values and theoretical one calculated by Equation (36) comparison of radial differential output capacitance.

Compared to the error distribution shown in Figure 8, the error CrX*_app.error_* is significantly decreased overall for the calculation expression of the CrX with third-order approximation, as shown in Figure 10. As the phase angle *β* varies from 0° to 90°, the error CrX*_app.error_* remains less than 0.4% for the ratio *α*/*g* = 0.1 and no more than 1.2% for *α*/*g* = 0.2. Figure 11 compares the simulated differential capacitance CrXf and theoretical counterparts CrX_tl3_ calculated with Equation (36). The phase angle *β* equals 45°, that is, the rotor has a displacement along the direction of y = x. It can be observed that the deviation between the value CrXf and the CrX_tl3_ is closer to the actual value.

In order to theoretically analyze the tilt error motion during the measurement, we considered tiny tilt displacement of a precision spindle and reasonable structural parameters of the sensor, that is, |*kρ*/*a*| < 1 for the parameter term *kρ*/*a* in Equation (15). The power series expansion with first-order approximation (Equation (38)) was also applied to express the integrand term in Equation (15):

(38) $${\mathrm{IGe}}_{\mathrm{tl}}=\frac{\rho }{a}\cdot \left[1+\frac{k}{a}\rho \cos(\varphi +\gamma )\right]$$

Correspondingly, the expression used to assess the approximate error is given as:

(39) $${\mathrm{IGe}}_{app.error}=\left|\frac{\mathrm{IGe}-{\mathrm{IGe}}_{\mathrm{tl}}}{\mathrm{IGe}}\right|$$

**Figure 12:**
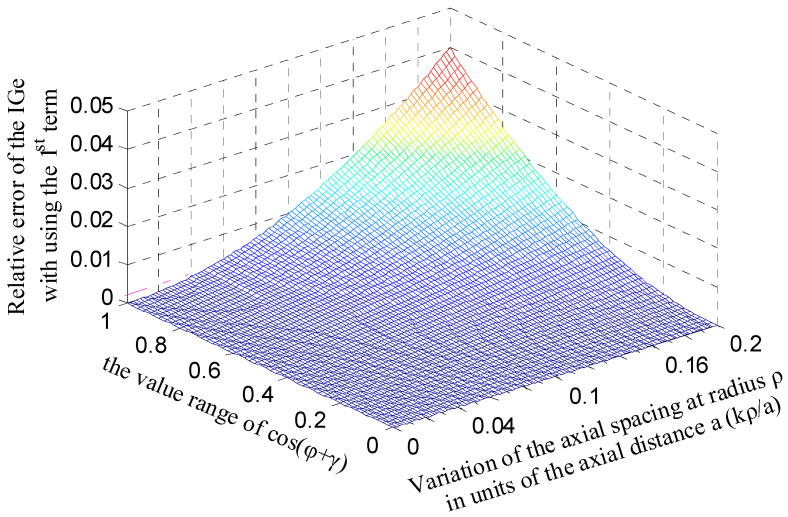
Error distribution of the first-order approximation of the integrand term in the capacitance expression of the end part electrode.

**Figure 13:**
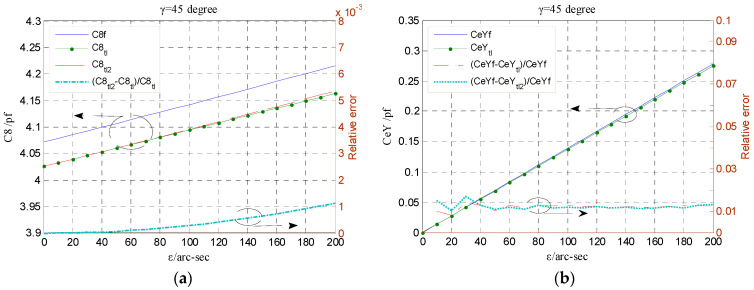
Comparison of the simulation values of the end part electrode capacitance and the theoretical one calculated by the integrand term with first-order and second-order approximation respectively: (**a**) Single electrode capacitance; (**b**) Differential output capacitance.

By considering the relative tiny tilt displacement of a precision spindle and reasonability of the sensor structural parameters, the variation range of the term *kρ*/*a* was set to be [0, 0.2]. As shown in Figure 12, the approximate error IGe*_app.error_* is relatively smaller over the range of the parameter terms cos(*φ* + *γ*) and *kρ*/*a*. According to the simulation model parameters of the sensor [16], the ratio of the term *kρ*/*a* is about 0.04 with the tilt displacement of 200 arc-sec; thus, the error IGe*_app.error_* remains no more than 0.2% within the whole range of the term cos(*φ* + *γ*), i.e., the entire phase region. Figure 13a compares the theoretical and simulated capacitance of the end part electrode. The theoretical capacitance is integrated by the integrand term with a second-order approximation (C8_tl2_). It can be observed that there is little difference between C8_tl2_ and the counterpart using first-order approximation (C8_tl_), which is also the same for the theoretical value of the CeY (the differential output capacitance of the EPEG) shown in Figure 13b. Besides, similar to the theoretical value of the CrX calculated by the expression with third-order approximation (see in Figure 11), the difference between the theoretical capacitance CeY_tl_ and the simulation one CeYf remains roughly unchanged relative to the simulation value, which indicates that the theoretical capacitance of the end part electrode solved by adopting IGe_tl_ as integrand can meet the design needs for relatively accurate results.

### 3.2. Influence of Fringe Effects

**Figure 14:**
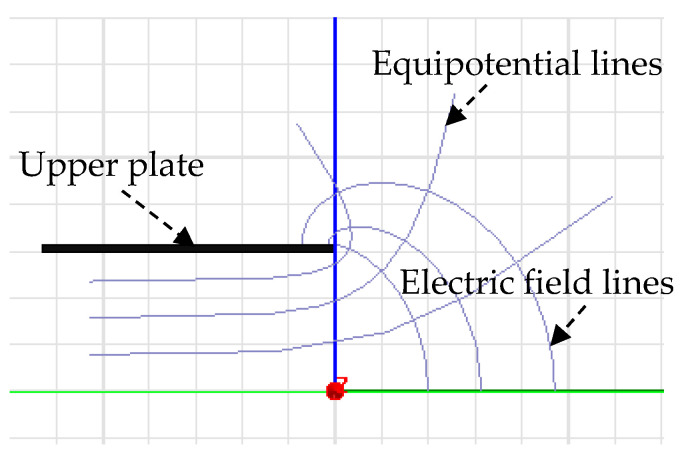
The fringe field distribution of a parallel plate capacitor with the gap 2π (the field lines between the two plates are symmetric, and thus only consider the distribution above the centerline).

Due to the existence of diverging electric fields in the plate edges in Figure 14, i.e., the “fringe effects” [19], the measured capacitance of a sensor contains some additional capacitance introduced by the fringe effects. As for the sensor electrode with finite dimensions, the variation in its output capacitance is relatively small, especially for the measurement of tiny displacement. As such, the influence of the fringe effects cannot be neglected.

**Figure 15:**
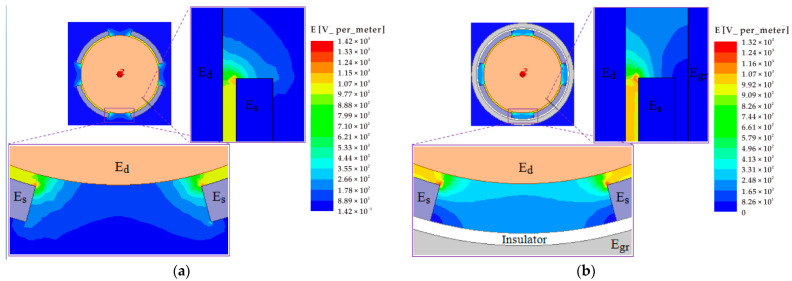
Electric field distribution between the electrode plates of the curved plate capacitor: (**a**) Without the *Egr*; (**b**) using the *Egr*.

Figure 15 shows the distribution of the electric field between the cylindrical excitation electrode (*E_d_*) and curved sensing electrodes (*E_s_*) for the fundamental configuration of the radial curved plate capacitor. It can be seen that the electric field concentration appears in the junction of the inner circle face of the curved plate and its side face. As shown in Figure 15a, a different intensity distribution of the electric field between the *E_d_* and the side face of the *E_s_* and its adjacent outer circular face formed along the radial direction. Once the equipotential guard ring (*Egr*) is employed outside the *E_s_*, the electric field distribution between the *E_d_* and the outer circular face of the *E_s_* and between the *E_d_* and local region of the side face near the outer circular face can be eliminated, as shown in Figure 15b. The *Egr* is also classically named Kelvin guard-ring.

**Figure 16:**
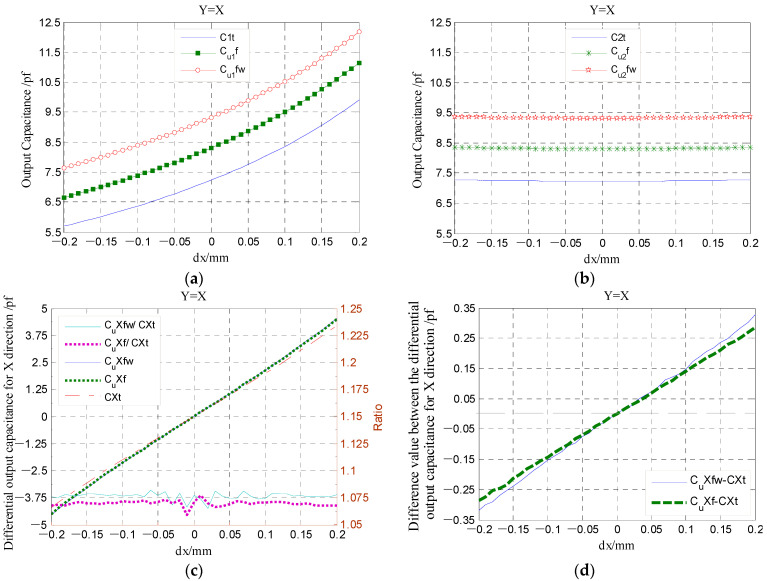
Simulation and theoretical value comparison for the output capacitance of the curved sensing electrodes *E_s_*: (**a**,**b**) The 1st, 2nd quadrant; (**c**,**d**) variation tendency of the differential output capacitance of the electrode and their relative difference value. The C*fw and C*f in the curve diagram denote the simulation values under without and with the *Egr,* respectively; the C*t is theoretical calculated value. The *dx* is defined as a component of the displacement *α* in the *x*-axis direction.

**Figure 17:**
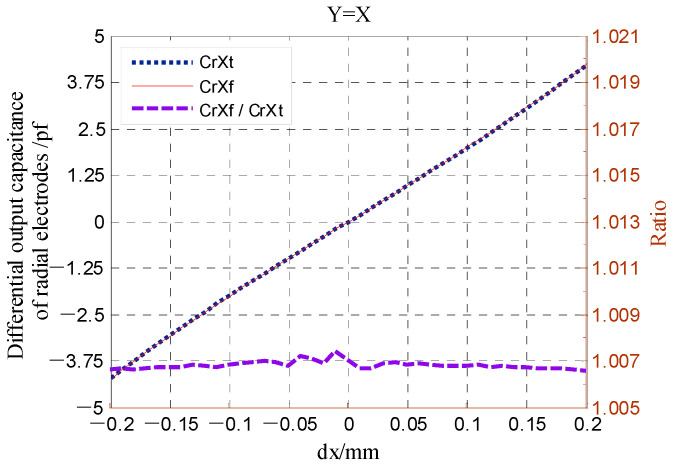
Simulation and theoretical values comparison of radial differential output capacitance of the T-type CS.

The variation in the electric field distribution is also reflected in the output capacitance of the *E_s_*, as illustrated in Figure 16. The use of the *Egr* can effectively reduce the additional capacitance introduced by the fringe effects (see Figure 16a,b). Moreover, the guard ring could decrease the variation in the additional capacitance with the reduction in radial spacing (see Figure 16d), which is helpful to improve the measurement accuracy and linearity. For the T-type CS, the *Egr* is distributed around the periphery of the sensing electrodes with a tiny gap (*λ* = 0.1 mm), forming a coplanar configuration between the *Egr* and the electrodes. Under the effect of the *Egr*, the amount of variation in the additional capacitance significantly decreased (about 10 times), and the corresponding nonlinear error reduced from 4.8% to 1.7%, as indicated in Figure 17.

### 3.3. Installation Errors of the Sensing Electrodes

The sensing electrodes of the T-type CS include the cylindrical and fan-shaped electrodes integrated into the REG and EPEG, respectively. These electrodes are fabricated by the FPCB process in one procedure, along with the equipotential guard ring, and the sensing electrodes have a fixed position relationship with the guard ring.

**Figure 18:**
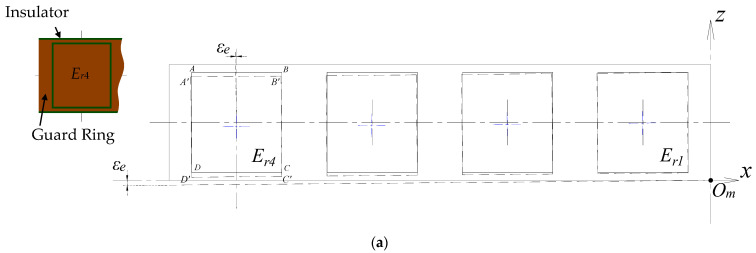

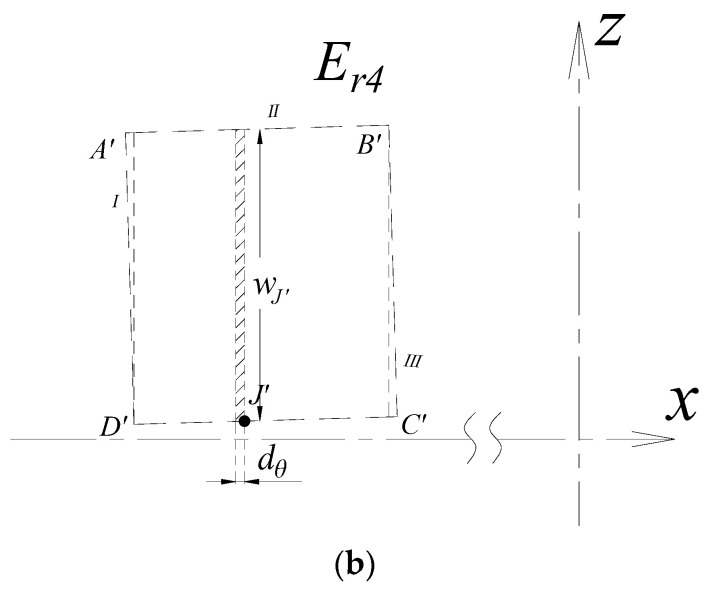
Tilt error analysis of the radial cylindrical electrode: (**a**) Local view of the REG structural model and schematic of the tilt error of cylindrical electrode (planar status); (**b**) geometric analysis model of single electrode. The *z*-axis is parallel to the medial axis of the stator, and the *x*-axis is parallel to axial reference of it.

The fabricated REG with planar form (as shown in Figure 18a, solid line box) is installed in the annular groove of the stator cylindrical bore by the surface mounting method. The coaxiality error, polar angle position error and tilt error (the axial boundary of the electrode relative to the axial reference of the stator) relative to the mounting reference were produced during the process. By utilizing the electrode measurement and sensor calibration method, the coaxiality and polar angle position error can be modified and removed, respectively. Thus, the analysis of the tilt error is the main focus for the cylindrical electrodes.

By spreading out the installed REG, as shown in Figure 18a, it can be seen that a certain tilt angle *ε_e_* is produced relative to the ideal position of the REG. The point *O_m_* is assumed to be the tilt point. The dash line box represents the position of the cylindrical electrodes, and the dash line segment represents the axial boundary of the REG. As mentioned above, the in-plane relationship between the cylindrical electrodes is fixed during the mounting process, and thus the tilt angle *ε_e_* is the tilt error of the electrodes.

As shown in Figure 18b, by taking a micro element with arc length *dθ* on the boundary *D′C′* of the electrode *E_r_*_4_, the axial length *w_J__′_* of the electrode at this location can be considered as the function of the polar angle *θ_J′_* at point *J′*. The solution region is vertically divided into three parts according to the constraint boundary of the length *w_J__′_*, and the corresponding calculation function can be expressed as follows:

(40) $$w_{I}=-k_{I}R\cdot \theta +b_{I}$$

(41) $$w_{II}=b_{II}$$

(42) $$w_{III}=-k_{III}R\cdot \theta +b_{III}$$

where *k_I_* = *k*_1_ + 1/*k*_1_, *k_III_* = −*k_I_*, *b_I_* = *b*_3_ − *b*_4_, *b_II_* = *b*_3_ − *b*_1_, *b_III_* = *b*_2_ − *b*_1_; *k*_1_ is the slope of the boundary *D′C′*; *b*_1_~*b*_4_ are the intercepts of the lines including *D′C′*, *C′B′*, *B′A′*, *D′A′*, respectively.

Referring to Equation (6), the approximate expression of the output capacitance of the electrode *E_r_*_4_ under the tilt angle *ε_e_* can be obtained:

(43) $${C4}^{\prime }=\varepsilon_{0}\varepsilon_{r}R\left[\int_{\theta_{D^{\prime }}}^{\theta_{A^{\prime }}}\frac{w_{I}}{g-\alpha \cos\left(\theta -\beta \right)}d\theta +\int_{\theta_{B^{\prime }}}^{\theta_{D^{\prime }}}\frac{w_{II}}{g-\alpha \cos\left(\theta -\beta \right)}d\theta +\int_{\theta_{C^{\prime }}}^{\theta_{B^{\prime }}}\frac{w_{III}}{g-\alpha \cos\left(\theta -\beta \right)}d\theta \right]$$

By considering the minor radial displacement of a precision spindle, at the condition |*α*/*g*| < 1, the power series expansion with neglection of the high-order term is applied to express the integrand term in Equation (43), and thus we have:

(44) $$C4^{\prime }=\frac{\varepsilon_{0}\varepsilon_{r}R}{g}\left\{\begin{array}{l} {\left[-k_{I}R\cdot \frac{\theta^{2}}{2}+b_{I}\theta -k_{I}R\left(\frac{\alpha }{g}\right)\left(\cos(\theta -\beta )+\left(\theta -\frac{b_{I}}{k_{I}R}\right)\cdot \sin(\theta -\beta )\right)-k_{I}R{\left(\frac{\alpha }{g}\right)}^{2}\left(\frac{\theta^{2}}{4}-\frac{b_{I}}{k_{I}R}\cdot \frac{\left(\theta -\beta \right)}{2}+\frac{\cos(2(\theta -\beta ))}{8}+\left(\theta -\frac{b_{I}}{k_{I}R}\right)\cdot \frac{\sin(2(\theta -\beta ))}{4}\right)\right]|}_{\theta_{D^{\prime }}}^{\theta_{A^{\prime }}}\\ +{\left[\frac{2b_{II}}{g-\alpha }\sqrt{\frac{g-\alpha }{g+\alpha }}\arctan\left(\sqrt{\frac{g+\alpha }{g-\alpha }}\tan\left(\frac{\theta -\beta }{2}\right)\right)\right]|}_{\theta_{B^{\prime }}}^{\theta_{D^{\prime }}}\\ +{\left[-k_{III}R\cdot \frac{\theta^{2}}{2}+b_{III}\theta -k_{III}R\left(\frac{\alpha }{g}\right)\left(\cos(\theta -\beta )+\left(\theta -\frac{b_{III}}{k_{III}R}\right)\cdot \sin(\theta -\beta )\right)-k_{III}R{\left(\frac{\alpha }{g}\right)}^{2}\left(\frac{\theta^{2}}{4}-\frac{b_{III}}{k_{III}R}\cdot \frac{\left(\theta -\beta \right)}{2}+\frac{\cos(2(\theta -\beta ))}{8}+\left(\theta -\frac{b_{III}}{k_{III}R}\right)\cdot \frac{\sin(2(\theta -\beta ))}{4}\right)\right]|}_{\theta_{C^{\prime }}}^{\theta_{B^{\prime }}} \end{array}\right\}$$

Similarly, the approximate expression of the output capacitance of the electrodes *E_r_*_3_~*E_r_*_1_ are derived as follows:

(45) $${C3}^{\prime }=\frac{\varepsilon_{0}\varepsilon_{r}R}{g}\left\{\begin{array}{l} {\left[-k_{2I}R\cdot \frac{\theta^{2}}{2}+b_{2I}\theta -k_{2I}R\left(\frac{\alpha }{g}\right)\left(\cos(\theta -\beta )+\left(\theta -\frac{b_{2I}}{k_{2I}R}\right)\cdot \sin(\theta -\beta )\right)-k_{2I}R{\left(\frac{\alpha }{g}\right)}^{2}\left(\frac{\theta^{2}}{4}-\frac{b_{2I}}{k_{2I}R}\cdot \frac{\left(\theta -\beta \right)}{2}+\frac{\cos(2(\theta -\beta ))}{8}+\left(\theta -\frac{b_{2I}}{k_{2I}R}\right)\cdot \frac{\sin(2(\theta -\beta ))}{4}\right)\right]|}_{\theta_{D2^{\prime }}}^{\theta_{A2^{\prime }}}\\ +{\left[\frac{2b_{2II}}{g-\alpha }\sqrt{\frac{g-\alpha }{g+\alpha }}\arctan\left(\sqrt{\frac{g+\alpha }{g-\alpha }}\tan\left(\frac{\theta -\beta }{2}\right)\right)\right]|}_{\theta_{B2^{\prime }}}^{\theta_{D2^{\prime }}}\\ +{\left[-k_{2III}R\cdot \frac{\theta^{2}}{2}+b_{2III}\theta -k_{2III}R\left(\frac{\alpha }{g}\right)\left(\cos(\theta -\beta )+\left(\theta -\frac{b_{2III}}{k_{2III}R}\right)\cdot \sin(\theta -\beta )\right)-k_{2III}R{\left(\frac{\alpha }{g}\right)}^{2}\left(\frac{\theta^{2}}{4}-\frac{b_{2III}}{k_{2III}R}\cdot \frac{\left(\theta -\beta \right)}{2}+\frac{\cos(2(\theta -\beta ))}{8}+\left(\theta -\frac{b_{2III}}{k_{2III}R}\right)\cdot \frac{\sin(2(\theta -\beta ))}{4}\right)\right]|}_{\theta_{C2^{\prime }}}^{\theta_{B2^{\prime }}} \end{array}\right\}$$

(46) $${C2}^{\prime }=\frac{\varepsilon_{0}\varepsilon_{r}R}{g}\left\{\begin{array}{l} {\left[-k_{3I}R\cdot \frac{\theta^{2}}{2}+b_{3I}\theta -k_{3I}R\left(\frac{\alpha }{g}\right)\left(\cos(\theta -\beta )+\left(\theta -\frac{b_{3I}}{k_{3I}R}\right)\cdot \sin(\theta -\beta )\right)-k_{3I}R{\left(\frac{\alpha }{g}\right)}^{2}\left(\frac{\theta^{2}}{4}-\frac{b_{3I}}{k_{3I}R}\cdot \frac{\left(\theta -\beta \right)}{2}+\frac{\cos(2(\theta -\beta ))}{8}+\left(\theta -\frac{b_{3I}}{k_{3I}R}\right)\cdot \frac{\sin(2(\theta -\beta ))}{4}\right)\right]|}_{\theta_{D3^{\prime }}}^{\theta_{A3^{\prime }}}\\ +{\left[\frac{2b_{3II}}{g-\alpha }\sqrt{\frac{g-\alpha }{g+\alpha }}\arctan\left(\sqrt{\frac{g+\alpha }{g-\alpha }}\tan\left(\frac{\theta -\beta }{2}\right)\right)\right]|}_{\theta_{B3^{\prime }}}^{\theta_{D3^{\prime }}}\\ +{\left[-k_{3III}R\cdot \frac{\theta^{2}}{2}+b_{3III}\theta -k_{3III}R\left(\frac{\alpha }{g}\right)\left(\cos(\theta -\beta )+\left(\theta -\frac{b_{3III}}{k_{3III}R}\right)\cdot \sin(\theta -\beta )\right)-k_{3III}R{\left(\frac{\alpha }{g}\right)}^{2}\left(\frac{\theta^{2}}{4}-\frac{b_{3III}}{k_{3III}R}\cdot \frac{\left(\theta -\beta \right)}{2}+\frac{\cos(2(\theta -\beta ))}{8}+\left(\theta -\frac{b_{3III}}{k_{3III}R}\right)\cdot \frac{\sin(2(\theta -\beta ))}{4}\right)\right]|}_{\theta_{C3^{\prime }}}^{\theta_{B3^{\prime }}} \end{array}\right\}$$

(47) $${C1}^{\prime }=\frac{\varepsilon_{0}\varepsilon_{r}R}{g}\left\{\begin{array}{l} {\left[-k_{4I}R\cdot \frac{\theta^{2}}{2}+b_{4I}\theta -k_{4I}R\left(\frac{\alpha }{g}\right)\left(\cos(\theta -\beta )+\left(\theta -\frac{b_{4I}}{k_{4I}R}\right)\cdot \sin(\theta -\beta )\right)-k_{4I}R{\left(\frac{\alpha }{g}\right)}^{2}\left(\frac{\theta^{2}}{4}-\frac{b_{4I}}{k_{4I}R}\cdot \frac{\left(\theta -\beta \right)}{2}+\frac{\cos(2(\theta -\beta ))}{8}+\left(\theta -\frac{b_{4I}}{k_{4I}R}\right)\cdot \frac{\sin(2(\theta -\beta ))}{4}\right)\right]|}_{\theta_{D4^{\prime }}}^{\theta_{A4^{\prime }}}\\ +{\left[\frac{2b_{4II}}{g-\alpha }\sqrt{\frac{g-\alpha }{g+\alpha }}\arctan\left(\sqrt{\frac{g+\alpha }{g-\alpha }}\tan\left(\frac{\theta -\beta }{2}\right)\right)\right]|}_{\theta_{B4^{\prime }}}^{\theta_{D4^{\prime }}}\\ +{\left[-k_{4III}R\cdot \frac{\theta^{2}}{2}+b_{4III}\theta -k_{4III}R\left(\frac{\alpha }{g}\right)\left(\cos(\theta -\beta )+\left(\theta -\frac{b_{4III}}{k_{4III}R}\right)\cdot \sin(\theta -\beta )\right)-k_{4III}R{\left(\frac{\alpha }{g}\right)}^{2}\left(\frac{\theta^{2}}{4}-\frac{b_{4III}}{k_{4III}R}\cdot \frac{\left(\theta -\beta \right)}{2}+\frac{\cos(2(\theta -\beta ))}{8}+\left(\theta -\frac{b_{4III}}{k_{4III}R}\right)\cdot \frac{\sin(2(\theta -\beta ))}{4}\right)\right]|}_{\theta_{C4^{\prime }}}^{\theta_{B4^{\prime }}} \end{array}\right\}$$

where, *k*_4*I*_ = *k*_3*I*_ = *k*_2*I*_ = *k_I_*, *k*_4*III*_ = *k*_3*III*_ = *k*_2*III*_ = *k_III_*, *b*_4*II*_ = *b*_3*II*_ = *b*_2*II*_ = *b_II_*, *b_iI_* = *b*_3_ − *b_i_*_4_, *b_iIII_* = *b_i_*_2_ − *b*_1_(*i* = 4, 3, 2); *b_i_*_2_, *b_i_*_4_ are the intercepts of the lines, including *Ci′Bi′*, *Di′Ai′* (*i* = 4, 3, 2), respectively; *i* represents the electrodes *E_r_*_1_~*E_r_*_3_ in turn.

Further, the influence of the tilt error *ε_e_* on the output capacitance of the cylindrical electrode (taking *E_r_*_4_ as an example) can be reflected by:

(48) $$C4_{tilt.error}=\frac{{C4}^{\prime }-C4}{C4}$$

**Figure 19:**
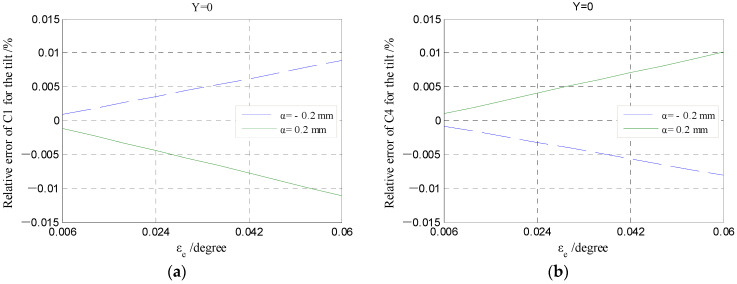

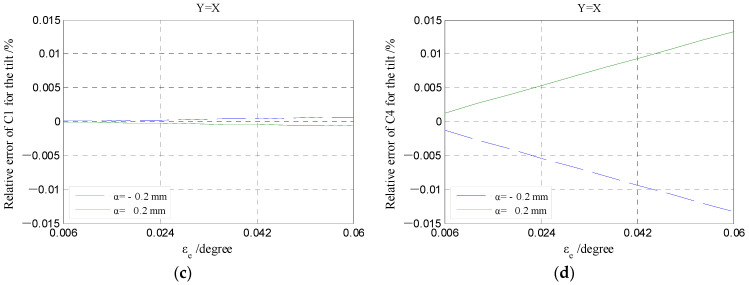
Relative error variations in the output capacitance of cylindrical electrodes under the different tilt angle *ε_e_*: (**a**,**c**) The 1st quadrant; (**b**,**d**) the 4th quadrant.

By referring to the structural design of the T-type CS, the range of the tilt angle *ε_e_* was set to be [0.006, 0.06] deg (about 200 arc-sec) in the numerical simulation using Matlab. A tiny circumferential displacement of the cylindrical electrode was produced due to the tilt angle *ε_e_*, which produces a relative error of the output capacitance under the radial displacement along the same direction. The dependence of the relative error on the angle *ε_e_* is shown in Figure 19. For the single electrode, the influence of the tilt angle *ε_e_* on the output capacitance is very small, with respect to the amplitude of the relative error.

**Figure 20:**
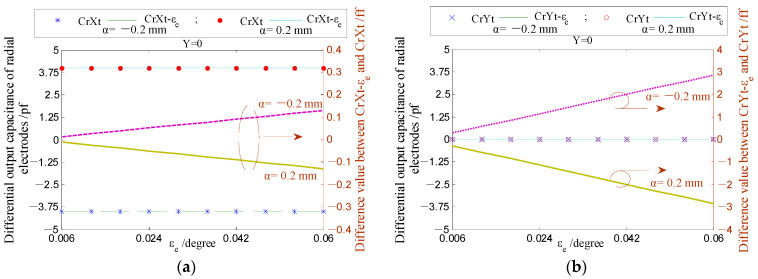

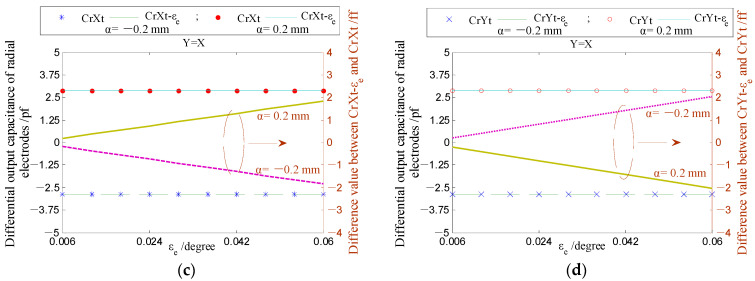
The changing of the radial differential output capacitance under the different tilt angle *ε_e_*: (**a**,**c**) The *X*-direction; (**b**,**d**) the *Y*-direction.

**Figure 21:**
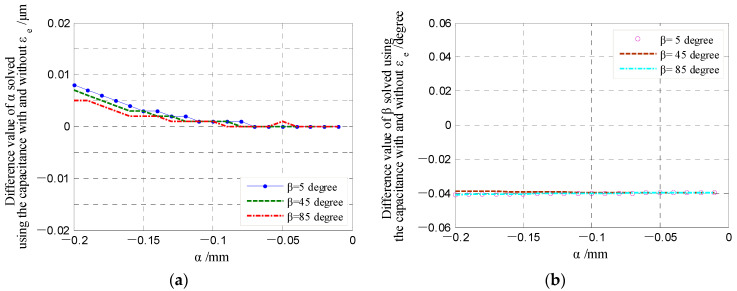
Comparison of the solved displacement parameters of the rotor under the different electrode poses: (**a**) Displacement *α*; (**b**) phase angle *β*.

Figure 20 presents the dependence of the differential capacitance on the tilt angle *ε_e_*. It can be known that the influence of the tilt angle *ε_e_* on the differential output capacitance of the REG is slightly larger. By considering the rotor of run-out in the given directions (*β* = 5°, 45°, 85°), the differential output capacitance (CrXt-*ε_e_* and CrYt-*ε_e_*) can be calculated by the capacitance of single electrodes under the angle *ε_e_*. The difference between the differential output capacitance under the angle *ε_e_* and the counterpart under an ideal position (CrXt and CrYt) could be transformed to the difference of displacement parameters, utilizing Equations (33) and (34). The difference in the displacement parameters (magnitude *α* and phase angle *β*) are shown in Figure 21. It can be known that the produced tilt error *ε_e_* of the cylindrical electrode only generates a certain deviation of solving the phase angle *β*, which is consistent in different displacement directions (brings about the overall phase advance or lag of the displacement trajectory); the effect of tilt error *ε_e_* on solving the radial displacement *α* can be neglected, especially for the measurement range of less than 0.1 mm.

**Figure 22:**
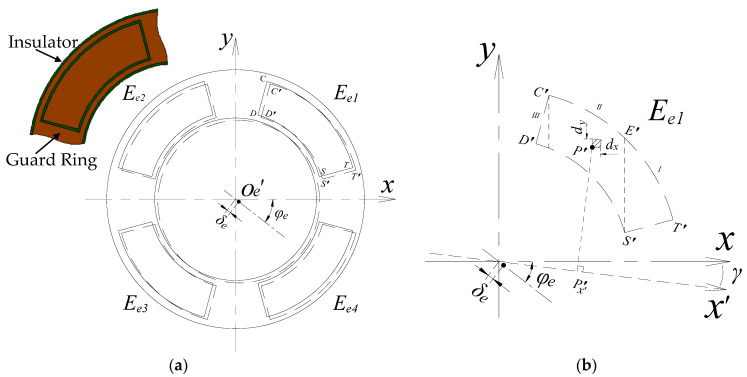
Coaxiality error analysis of the end part fan-shaped electrode: (**a**) Local view of the EPEG structural model and schematic of the coaxiality error of fan-shaped electrode (planar status); (**b**) geometric analysis model of single electrode.

The fabricated EPEG was also in planar form (as shown in Figure 22a, solid line box) and installed in the annular groove located at the end part of the stator by the surface mounting method. The coaxiality error, polar angle position error and parallelism error (the electrode plane relative to the axial reference of the stator) relative to the mounting reference were produced during the process. For the parallelism error, it can be modified by the electrode measurement. Thus, the analysis of the fan-shaped electrodes is emphasized on the coaxiality error and polar angle position error.

Due to the position error between the shape boundary of EPEG and the sensing electrodes and the manipulation precision, there may be an eccentricity between the geometric center *O_e_′* of the fan-shaped electrodes in the EPEG and the medial axis of the stator (the origin position of the coordinate system). As shown in Figure 22a, *δ_e_* denotes the magnitude of the eccentricity, and *φ_e_* is the phase angle. *δ_e_* is the coaxiality error of the fan-shaped electrode.

By taking the electrode *E_e_*_1_ as an example, the region confined by the points *C′D′S′T′* (dash line) represents the position of *E_e_*_1_ under the coaxiality error *δ_e_*. As shown in Figure 22b, the micro-plane element ΔB_P′_ (oblique line filled) at any point *P′* is taken as the solving unit, and the area of this element is denoted as Δ*S_P′_*. Then, the Δ*S_P′_* is approximately given by:

(49) $$\Delta S_{P^{\prime }}=dx\cdot dy$$

The projection point of *P′* on the *x′*-axis is the point *P′_x′_*. By referring to Equation (13), the expression for calculating the spacing *t′* at this point could be derived as:

(50) $$t^{\prime }=a-k(x\cos\gamma -y\sin\gamma )$$

Correspondingly, the capacitance of the plane parallel capacitor composed of the micro-planes ΔB_P′_ and ΔA_Q′_ is:

(51) $$\Delta C_{e}{}^{\prime }=\frac{\varepsilon_{0}\varepsilon_{r}\Delta S_{P^{\prime }}}{t^{\prime }}=\varepsilon_{0}\varepsilon_{r}\frac{dx\cdot dy}{a-k(x\cos\gamma -y\sin\gamma )}$$

Through the integration of Equation (51) over the area of the fan-shaped electrode, the output capacitance of the electrode *E_e_*_1_ is approximately expressed as:

(52) $$C5^{\prime }=\iint_{D}\Delta C_{e}{}^{\prime }=\varepsilon_{0}\varepsilon_{r}\iint_{D}\frac{dxdy}{a-k(x\cos\gamma -y\sin\gamma )}$$

where the integral domain *D* is a closed region confined by the boundary curves of the fan-shaped electrode, which can be divided into three sub-regions, i.e., *D_I_*, *D_II_* and *D_III_*. These three sub-regions can be expressed as follows:

$$\begin{array}{c} D_{III}=\left\{\left(x,y\right)|\sqrt{\rho_{1}^{2}-{\left(x-x_{B}\right)}^{2}}+y_{B}\leq y\leq k_{C^{\prime }}x+b_{C^{\prime }},{D^{\prime }}_{x}\leq x\leq {C^{\prime }}_{x}\right\},\\ D_{II}=\left\{\left(x,y\right)|\sqrt{\rho_{1}^{2}-{\left(x-x_{B}\right)}^{2}}+y_{B}\leq y\leq \sqrt{\rho_{2}^{2}-{\left(x-x_{B}\right)}^{2}}+y_{B},{C^{\prime }}_{x}\leq x\leq {E^{\prime }}_{x}\right\},\\ D_{I}=\left\{\left(x,y\right)|k_{S^{\prime }}x+b_{S^{\prime }}\leq y\leq \sqrt{\rho_{2}^{2}-{\left(x-x_{B}\right)}^{2}}+y_{B},{E^{\prime }}_{x}\leq x\leq {T^{\prime }}_{x}\right\}. \end{array}$$

where *x_B_* and *y_B_* are the coordinates of the geometric center *O_e_′*; *D′_x_*, *C′_x_*, *E′_x_* and *T′_x_* represent the abscissas of the corresponding points, respectively.

Equation (52) can be rewritten as:

(53) $$C5^{\prime }=\frac{\varepsilon_{0}\varepsilon_{r}}{a}\iint_{D}\frac{dxdy}{1-\frac{k}{a}(x\cos\gamma -y\sin\gamma )}$$

As for multivariate function, it can be expanded to power series under certain conditions by analogy with the univariate power series expansion [20]. By considering the tiny tilt displacement of a precision spindle and reasonable structural parameters of the sensor, there is generally |(*k*/*a*)∙(*x*cos(*γ*) − *y*sin(*γ*))| << 1, the power series expansion with neglecting of a high-order term is applied to express the integrand term in Equation (53). Then, it can be expressed as:

(54) $$C5^{\prime }=\frac{\varepsilon_{0}\varepsilon_{r}}{a}\iint_{D}\left[1+\frac{k}{a}(x\cos\gamma -y\sin\gamma )\right]dxdy$$

By calculating Equation (54) within the integral sub-domains *D_I_*, *D_II_* and *D_III_*, we have:

(55) $$\begin{array}{l} C5^{\prime }=\frac{\varepsilon_{0}\varepsilon_{r}}{a}\left\{\begin{array}{l} \int_{{D^{\prime }}_{x}}^{{C^{\prime }}_{x}}dx\int_{\sqrt{\rho_{1}^{2}-{\left(x-x_{B}\right)}^{2}}+y_{B}}^{k_{C^{\prime }}x+b_{C^{\prime }}}\left[1+\frac{k}{a}(x\cos\gamma -y\sin\gamma )\right]dy+\int_{{C^{\prime }}_{x}}^{{E^{\prime }}_{x}}dx\int_{\sqrt{\rho_{1}^{2}-{\left(x-x_{B}\right)}^{2}}+y_{B}}^{\sqrt{\rho_{2}^{2}-{\left(x-x_{B}\right)}^{2}}+y_{B}}\left[1+\frac{k}{a}(x\cos\gamma -y\sin\gamma )\right]dy\\ +\int_{{E^{\prime }}_{x}}^{{T^{\prime }}_{x}}dx\int_{k_{S^{\prime }}x+b_{S^{\prime }}}^{\sqrt{\rho_{2}^{2}-{\left(x-x_{B}\right)}^{2}}+y_{B}}\left[1+\frac{k}{a}(x\cos\gamma -y\sin\gamma )\right]dy \end{array}\right\}\\ \;\;\;=\frac{\varepsilon_{0}\varepsilon_{r}}{a}\left\{\begin{array}{l} {\left[\begin{array}{l} \left(k_{C^{\prime }}+\mu b_{C^{\prime }}-\nu k_{C^{\prime }}b_{C^{\prime }}-\mu y_{B}+\nu x_{B})\frac{x^{2}}{2}+(\mu k_{C^{\prime }}-\frac{\nu }{2}k_{C^{\prime }}^{2}-\frac{\nu }{2})\frac{x^{3}}{3}-(1-\nu y_{B}+\mu x_{B})\cdot (\frac{x-x_{B}}{2}\sqrt{\rho_{1}^{2}-{\left(x-x_{B}\right)}^{2}}+\frac{\rho_{1}^{2}}{2}\arcsin\frac{x-x_{B}}{\rho_{1}}\right)\\ +\frac{\mu }{3}\sqrt{{\left(\rho_{1}^{2}-{\left(x-x_{B}\right)}^{2}\right)}^{3}}+((b_{C^{\prime }}-y_{B})-\frac{\nu }{2}(b_{C^{\prime }}^{2}-\rho_{1}^{2})-\frac{\nu }{2}(x_{B}^{2}-y_{B}^{2}))\cdot x \end{array}\right]|}_{{D^{\prime }}_{x}}^{{C^{\prime }}_{x}}\\ +{\left[\begin{array}{l} \left(1-\nu y_{B}+\mu x_{B})\cdot (\frac{x-x_{B}}{2}(\sqrt{\rho_{2}^{2}-{\left(x-x_{B}\right)}^{2}}-\sqrt{\rho_{1}^{2}-{\left(x-x_{B}\right)}^{2}})+\frac{\rho_{2}^{2}}{2}\arcsin\frac{x-x_{B}}{\rho_{2}}-\frac{\rho_{1}^{2}}{2}\arcsin\frac{x-x_{B}}{\rho_{1}}\right)\\ +\frac{\mu }{3}(\sqrt{{\left(\rho_{1}^{2}-{\left(x-x_{B}\right)}^{2}\right)}^{3}}-\sqrt{{\left(\rho_{2}^{2}-{\left(x-x_{B}\right)}^{2}\right)}^{3}})-\frac{\nu }{2}(\rho_{2}^{2}-\rho_{1}^{2})\cdot x \end{array}\right]|}_{{C^{\prime }}_{x}}^{{E^{\prime }}_{x}}\\ +{\left[\begin{array}{l} \left(\mu y_{B}-\nu x_{B}+\nu k_{S^{\prime }}b_{S^{\prime }}-\mu b_{S^{\prime }}-k_{S^{\prime }})\frac{x^{2}}{2}+(\frac{\nu }{2}+\frac{\nu }{2}k_{S^{\prime }}^{2}-\mu k_{S^{\prime }})\frac{x^{3}}{3}+(1-\nu y_{B}+\mu x_{B})\cdot (\frac{x-x_{B}}{2}\sqrt{\rho_{2}^{2}-{\left(x-x_{B}\right)}^{2}}+\frac{\rho_{2}^{2}}{2}\arcsin\frac{x-x_{B}}{\rho_{2}}\right)\\ -\frac{\mu }{3}\sqrt{{\left(\rho_{2}^{2}-{\left(x-x_{B}\right)}^{2}\right)}^{3}}+((y_{B}-b_{S^{\prime }})-\frac{\nu }{2}(\rho_{2}^{2}-b_{S^{\prime }}^{2})-\frac{\nu }{2}(y_{B}^{2}-x_{B}^{2}))\cdot x \end{array}\right]|}_{{E^{\prime }}_{x}}^{{T^{\prime }}_{x}} \end{array}\right\} \end{array}$$

Similarly, the approximate expressions of the output capacitance of the electrodes *E_e_*_2_~*E_e_*_4_ can be derived as follows:

(56) $$C6^{\prime }=\frac{\varepsilon_{0}\varepsilon_{r}}{a}\left\{\begin{array}{l} {\left[\begin{array}{l} \left(k_{{C^{\prime }}_{1}}+\mu b_{{C^{\prime }}_{1}}-\nu k_{{C^{\prime }}_{1}}b_{{C^{\prime }}_{1}}-\mu y_{B}+\nu x_{B})\frac{x^{2}}{2}+(\mu k_{{C^{\prime }}_{1}}-\frac{\nu }{2}k_{{C^{\prime }}_{1}}^{2}-\frac{\nu }{2})\frac{x^{3}}{3}-(1-\nu y_{B}+\mu x_{B})\cdot (\frac{x-x_{B}}{2}\sqrt{\rho_{1}^{2}-{\left(x-x_{B}\right)}^{2}}+\frac{\rho_{1}^{2}}{2}\arcsin\frac{x-x_{B}}{\rho_{1}}\right)\\ +\frac{\mu }{3}\sqrt{{\left(\rho_{1}^{2}-{\left(x-x_{B}\right)}^{2}\right)}^{3}}+((b_{{C^{\prime }}_{1}}-y_{B})-\frac{\nu }{2}(b_{{C^{\prime }}_{1}}^{2}-\rho_{1}^{2})-\frac{\nu }{2}(x_{B}^{2}-y_{B}^{2}))\cdot x \end{array}\right]|}_{{C^{\prime }}_{1x}}^{{D^{\prime }}_{1x}}\\ +{\left[\begin{array}{l} \left(1-\nu y_{B}+\mu x_{B})\cdot (\frac{x-x_{B}}{2}(\sqrt{\rho_{2}^{2}-{\left(x-x_{B}\right)}^{2}}-\sqrt{\rho_{1}^{2}-{\left(x-x_{B}\right)}^{2}})+\frac{\rho_{2}^{2}}{2}\arcsin\frac{x-x_{B}}{\rho_{2}}-\frac{\rho_{1}^{2}}{2}\arcsin\frac{x-x_{B}}{\rho_{1}}\right)\\ +\frac{\mu }{3}(\sqrt{{\left(\rho_{1}^{2}-{\left(x-x_{B}\right)}^{2}\right)}^{3}}-\sqrt{{\left(\rho_{2}^{2}-{\left(x-x_{B}\right)}^{2}\right)}^{3}})-\frac{\nu }{2}(\rho_{2}^{2}-\rho_{1}^{2})\cdot x \end{array}\right]|}_{{E^{\prime }}_{1x}}^{{C^{\prime }}_{1x}}\\ +{\left[\begin{array}{l} \left(\mu y_{B}-\nu x_{B}+\nu k_{{S^{\prime }}_{1}}b_{{S^{\prime }}_{1}}-\mu b_{{S^{\prime }}_{1}}-k_{{S^{\prime }}_{1}})\frac{x^{2}}{2}+(\frac{\nu }{2}+\frac{\nu }{2}k_{{S^{\prime }}_{1}}^{2}-\mu k_{{S^{\prime }}_{1}})\frac{x^{3}}{3}+(1-\nu y_{B}+\mu x_{B})\cdot (\frac{x-x_{B}}{2}\sqrt{\rho_{2}^{2}-{\left(x-x_{B}\right)}^{2}}+\frac{\rho_{2}^{2}}{2}\arcsin\frac{x-x_{B}}{\rho_{2}}\right)\\ -\frac{\mu }{3}\sqrt{{\left(\rho_{2}^{2}-{\left(x-x_{B}\right)}^{2}\right)}^{3}}+((y_{B}-b_{{S^{\prime }}_{1}})-\frac{\nu }{2}(\rho_{2}^{2}-b_{{S^{\prime }}_{1}}^{2})-\frac{\nu }{2}(y_{B}^{2}-x_{B}^{2}))\cdot x \end{array}\right]|}_{{T^{\prime }}_{1x}}^{{E^{\prime }}_{1x}} \end{array}\right\}$$

(57) $$C7^{\prime }=\frac{\varepsilon_{0}\varepsilon_{r}}{a}\left\{\begin{array}{l} {\left[\begin{array}{l} \left(\mu y_{B}-\nu x_{B}+\nu k_{C^{\prime }}b_{C^{\prime }}-\mu b_{C^{\prime }}-k_{C^{\prime }})\frac{x^{2}}{2}+(\frac{\nu }{2}+\frac{\nu }{2}k_{C^{\prime }}^{2}-\mu k_{C^{\prime }})\frac{x^{3}}{3}-(1-\nu y_{B}+\mu x_{B})\cdot (\frac{x-x_{B}}{2}\sqrt{\rho_{1}^{2}-{\left(x-x_{B}\right)}^{2}}+\frac{\rho_{1}^{2}}{2}\arcsin\frac{x-x_{B}}{\rho_{1}}\right)\\ +\frac{\mu }{3}\sqrt{{\left(\rho_{1}^{2}-{\left(x-x_{B}\right)}^{2}\right)}^{3}}+((y_{B}-b_{C^{\prime }})-\frac{\nu }{2}(\rho_{1}^{2}-b_{C^{\prime }}^{2})-\frac{\nu }{2}(y_{B}^{2}-x_{B}^{2}))\cdot x \end{array}\right]|}_{{C^{\prime }}_{2x}}^{{D^{\prime }}_{2x}}\\ +{\left[\begin{array}{l} \left(1-\nu y_{B}+\mu x_{B})\cdot (\frac{x-x_{B}}{2}(\sqrt{\rho_{2}^{2}-{\left(x-x_{B}\right)}^{2}}-\sqrt{\rho_{1}^{2}-{\left(x-x_{B}\right)}^{2}})+\frac{\rho_{2}^{2}}{2}\arcsin\frac{x-x_{B}}{\rho_{2}}-\frac{\rho_{1}^{2}}{2}\arcsin\frac{x-x_{B}}{\rho_{1}}\right)\\ +\frac{\mu }{3}(\sqrt{{\left(\rho_{1}^{2}-{\left(x-x_{B}\right)}^{2}\right)}^{3}}-\sqrt{{\left(\rho_{2}^{2}-{\left(x-x_{B}\right)}^{2}\right)}^{3}})-\frac{\nu }{2}(\rho_{1}^{2}-\rho_{2}^{2})\cdot x \end{array}\right]|}_{{E^{\prime }}_{2x}}^{{C^{\prime }}_{2x}}\\ +{\left[\begin{array}{l} \left(k_{S^{\prime }}+\mu b_{S^{\prime }}-\nu k_{S^{\prime }}b_{S^{\prime }}-\mu y_{B}+\nu x_{B})\frac{x^{2}}{2}+(\mu k_{S^{\prime }}-\frac{\nu }{2}k_{S^{\prime }}^{2}-\frac{\nu }{2})\frac{x^{3}}{3}+(1-\nu y_{B}+\mu x_{B})\cdot (\frac{x-x_{B}}{2}\sqrt{\rho_{2}^{2}-{\left(x-x_{B}\right)}^{2}}+\frac{\rho_{2}^{2}}{2}\arcsin\frac{x-x_{B}}{\rho_{2}}\right)\\ -\frac{\mu }{3}\sqrt{{\left(\rho_{2}^{2}-{\left(x-x_{B}\right)}^{2}\right)}^{3}}+((b_{S^{\prime }}-y_{B})-\frac{\nu }{2}(b_{S^{\prime }}^{2}-\rho_{2}^{2})-\frac{\nu }{2}(x_{B}^{2}-y_{B}^{2}))\cdot x \end{array}\right]|}_{{T^{\prime }}_{2x}}^{{E^{\prime }}_{2x}} \end{array}\right\}$$

(58) $$C8^{\prime }=\frac{\varepsilon_{0}\varepsilon_{r}}{a}\left\{\begin{array}{l} {\left[\begin{array}{l} \left(\mu y_{B}-\nu x_{B}+\nu k_{{C^{\prime }}_{1}}b_{{C^{\prime }}_{1}}-\mu b_{{C^{\prime }}_{1}}-k_{{C^{\prime }}_{1}})\frac{x^{2}}{2}+(\frac{\nu }{2}+\frac{\nu }{2}k_{{C^{\prime }}_{1}}^{2}-\mu k_{{C^{\prime }}_{1}})\frac{x^{3}}{3}-(1-\nu y_{B}+\mu x_{B})\cdot (\frac{x-x_{B}}{2}\sqrt{\rho_{1}^{2}-{\left(x-x_{B}\right)}^{2}}+\frac{\rho_{1}^{2}}{2}\arcsin\frac{x-x_{B}}{\rho_{1}}\right)\\ +\frac{\mu }{3}\sqrt{{\left(\rho_{1}^{2}-{\left(x-x_{B}\right)}^{2}\right)}^{3}}+((y_{B}-b_{{C^{\prime }}_{1}})-\frac{\nu }{2}(\rho_{1}^{2}-b_{{C^{\prime }}_{1}}^{2})-\frac{\nu }{2}(y_{B}^{2}-x_{B}^{2}))\cdot x \end{array}\right]|}_{{D^{\prime }}_{3x}}^{{C^{\prime }}_{3x}}\\ +{\left[\begin{array}{l} \left(1-\nu y_{B}+\mu x_{B})\cdot (\frac{x-x_{B}}{2}(\sqrt{\rho_{2}^{2}-{\left(x-x_{B}\right)}^{2}}-\sqrt{\rho_{1}^{2}-{\left(x-x_{B}\right)}^{2}})+\frac{\rho_{2}^{2}}{2}\arcsin\frac{x-x_{B}}{\rho_{2}}-\frac{\rho_{1}^{2}}{2}\arcsin\frac{x-x_{B}}{\rho_{1}}\right)\\ +\frac{\mu }{3}(\sqrt{{\left(\rho_{1}^{2}-{\left(x-x_{B}\right)}^{2}\right)}^{3}}-\sqrt{{\left(\rho_{2}^{2}-{\left(x-x_{B}\right)}^{2}\right)}^{3}})-\frac{\nu }{2}(\rho_{1}^{2}-\rho_{2}^{2})\cdot x \end{array}\right]|}_{{C^{\prime }}_{3x}}^{{E^{\prime }}_{3x}}\\ +{\left[\begin{array}{l} \left(k_{{S^{\prime }}_{1}}+\mu b_{{S^{\prime }}_{1}}-\nu k_{{S^{\prime }}_{1}}b_{{S^{\prime }}_{1}}-\mu y_{B}+\nu x_{B})\frac{x^{2}}{2}+(\mu k_{{S^{\prime }}_{1}}-\frac{\nu }{2}k_{{S^{\prime }}_{1}}^{2}-\frac{\nu }{2})\frac{x^{3}}{3}+(1-\nu y_{B}+\mu x_{B})\cdot (\frac{x-x_{B}}{2}\sqrt{\rho_{2}^{2}-{\left(x-x_{B}\right)}^{2}}+\frac{\rho_{2}^{2}}{2}\arcsin\frac{x-x_{B}}{\rho_{2}}\right)\\ -\frac{\mu }{3}\sqrt{{\left(\rho_{2}^{2}-{\left(x-x_{B}\right)}^{2}\right)}^{3}}+((b_{{S^{\prime }}_{1}}-y_{B})-\frac{\nu }{2}(b_{{S^{\prime }}_{1}}^{2}-\rho_{2}^{2})-\frac{\nu }{2}(x_{B}^{2}-y_{B}^{2}))\cdot x \end{array}\right]|}_{{E^{\prime }}_{3x}}^{{T^{\prime }}_{3x}} \end{array}\right\}$$

where *D′_ix_*, *C′_ix_*, *E′_ix_* and *T′_ix_*_(*i*=1,2,3)_ represent the abscissas of boundary points of the electrodes in the 2nd to 4th quadrant, respectively; $k_{{C^{\prime }}_{1}}=-k_{C^{\prime }},\;k_{{S^{\prime }}_{1}}=-k_{S^{\prime }}$; *μ* = *k*cos(*γ*)/*a*, *ν* = *k*sin(*γ*)/*a*.

Further, the expression used to assess the influence of the coaxiality error on the output capacitance of the fan-shaped electrode (taking *E_e_*_1_ as an example) is given as:

(59) $$C5_{ecc.error}=\frac{C5^{\prime }-C5}{C5}$$

**Figure 23:**
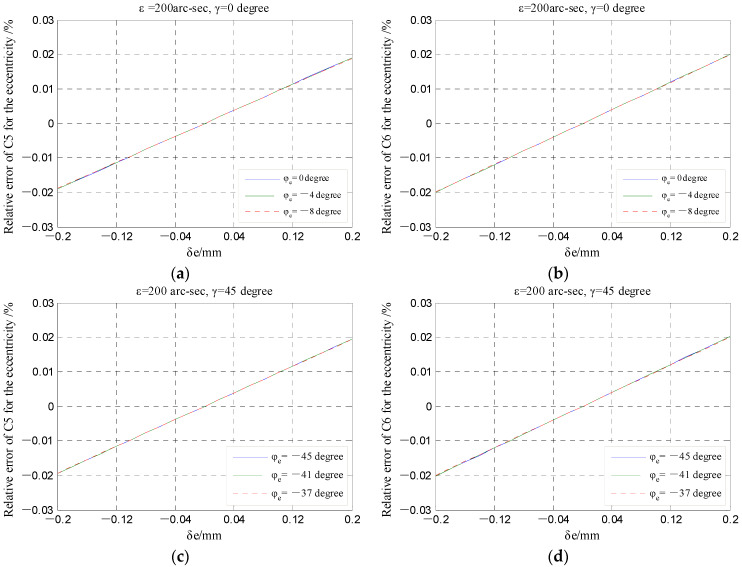
Relative error variations in the output capacitance of fan-shaped electrodes under the different coaxiality error *δ_e_*: (**a**,**c**) The 1st quadrant; (**b**,**d**) the 2nd quadrant.

In the numerical simulation using Matlab, the range of the coaxiality error *δ_e_* is set to be [−0.2, 0.2] mm, and the variation quantity of the phase angle *φ_e_* is 8 degrees referring to the sensor structural design. Figure 23 shows the relative error variation in the output capacitance of fan-shaped electrodes due to the error *δ_e_* under different phase angles *φ_e_*. For each fan-shaped electrode, the relative error of output capacitance increases with the increase in the error *δ_e_*. It should be pointed out that the magnitude of the relative error is quite small within the whole value range of the error *δ_e_*. Moreover, the influence of the phase angle *φ_e_* variation in the relative error is also very limited.

**Figure 24:**
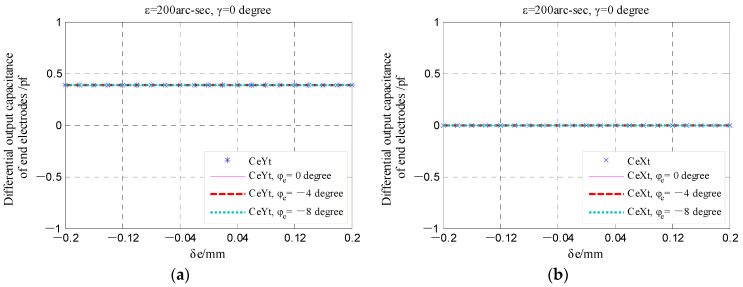

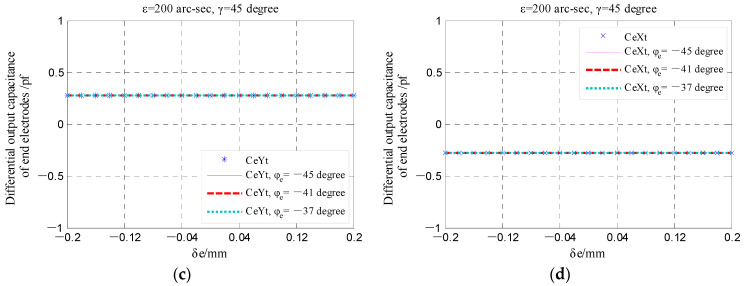
The changing of the end part differential output capacitance under the different coaxiality error *δ_e_*: (**a**,**c**) The component around the *y*-axis; (**b**,**d**) the component around the *x*-axis.

From Equations (27) and (28), it can be known that the tilt displacement *ε* and yaw angle *γ* are obtained based on the calculated differential output capacitance of the EPEG (CeY, CeX). As shown in Figure 24, under different yaw angle *γ*, the produced coaxiality error *δ_e_* and phase angle *φ_e_* do not cause the changing of the capacitance CeY and CeX, which indicates that the measurement of the rotor tilt displacement is basically not affected by the coaxiality error of fan-shaped electrode.

The polar angle position error of the fan-shaped electrodes equals each other and does not change the increment of the electrode polar angle. Thus, the effect of the polar angle position error on the yaw angle *γ* measurement can be eliminated by the sensor calibration method.

## 4. Conclusions

The main error sources for the error motion measurement of a precision shafting using a T-type capacitive sensor (CS) were investigated in this paper. Firstly, we analyzed the theoretical modeling error due to the approximate simplification for the capacitance expressions of each sensing electrode. The approximate error of the theoretical calculations for the radial error motion measurement could be reduced under small ratios *α*/(*R* − *g*) and *α*/*g*. The introduction of the third-order term in the approximate expressions of the CrX and CrY could significantly reduce the approximate error from 5.5% to 1.2% without sacrificing the measuring range and sensitivity. For the theoretical model for the tilt error motion measurement, the theoretical capacitance of the end part electrodes was solved by adopting IGe_tl_ (first-order approximation) as integrand can achieve relatively accurate results with an approximate error lower than 0.2%.

Subsequently, the 3D-FEA results indicate that for the T-type CS, the coplanar configuration of the sensing electrodes and *Egr* with a tiny gap (0.1 mm) could significantly decrease the amount of variation in the additional capacitance introduced by fringe effects and reduce the corresponding nonlinear error from 4.8% to 1.7%.

Finally, among the electrode installation errors, we emphatically analyzed the tilt error of the cylindrical electrode and coaxiality error of the fan-shaped electrode. Numerical simulation was carried out based on the measurement model with the installation errors. The simulation results show that the tilt error only generates a certain deviation of solving the phase angle *β* (consistent in different displacement directions). The effect of the tilt error on solving the radial displacement *α* can be neglected, especially for the measurement range of less than 0.1 mm. Additionally, the measurement of the rotor tilt displacement is basically not affected by the coaxiality error of the fan-shaped electrode.
